# Rare Germline Variants in Key Pathways Contribute to Hepatocellular Carcinoma Risk in Hispanic Individuals

**DOI:** 10.3390/cancers18152428

**Published:** 2026-07-28

**Authors:** Xiangnan Li, Yanhong Liu, Spiridon Tsavachidis, Priya B. Shetty, Amit G. Singal, Ruben Hernaez, Sumeet K. Asrani, Saira Khaderi, Hashem B. El-Serag, Aaron P. Thrift

**Affiliations:** 1 Section of Epidemiology and Population Sciences, Department of Medicine, Baylor College of Medicine, Houston, TX 77030, USAyl10@bcm.edu (Y.L.);; 2 Dan L Duncan Comprehensive Cancer Center, Department of Medicine, Baylor College of Medicine, Houston, TX 77030, USA; 3 Division of Digestive and Liver Diseases, Department of Medicine, UT Southwestern Medical Center, Dallas, TX 75390, USA; 4 Section of Gastroenterology and Hepatology, Department of Medicine, Baylor College of Medicine, Houston, TX 77030, USA; 5 Center for Innovations in Quality, Effectiveness and Safety (IQuESt), Michael E. DeBakey Veterans Affairs Medical Center, Houston, TX 77030, USA; 6 Division of Hepatology, Baylor University Medical Center, Dallas, TX 75246, USA

**Keywords:** hepatocellular carcinoma, genetic susceptibility, rare variant, Hispanic

## Abstract

**Background:** Hepatocellular carcinoma (HCC) is one of the leading causes of cancer-related death worldwide. In the U.S., HCC incidence is highest among Hispanic populations. With the exception of well-known *PNPLA3* variants, the genetic risk factors underlying HCC in Hispanic individuals, particularly rare germline variants, are largely unexplored. **Methods:** We performed whole-exome sequencing in 719 Hispanic Americans, including 455 HCC cases and 264 controls. We analyzed rare to low frequency, putatively deleterious variants in 86 genes previously implicated in HCC susceptibility. Genetic associations were assessed at single-variant, gene, and cumulative multi-variant burden levels using Fisher’s exact tests and weighted burden tests. **Results:** We identified 29 rare deleterious variants across 16 genes enriched in HCC cases compared with controls. Single-variant tests identified statistically robust associations in pathways related to lipids metabolism, *TM6SF2* R138W, immune regulation, *HLA-DRB1* W38X, and Wnt signaling pathway, *WNT9A* R375H. Exploratory analyses showed that *TM6SF2* R138W was further enriched among cases with alcohol and metabolic dysfunction-associated steatotic liver disease. Gene-based burden tests revealed five associated genes, including *TM6SF2* and *HLA-DRB1*. A strong dose-effect relationship was observed, with HCC risk increasing ~4-fold per candidate variant carried (odds ratio, 3.84; 95% confidence interval, 2.40–6.53). **Conclusions:** Rare deleterious germline variants across lipid metabolism and immune pathways contribute to increased HCC risk in Hispanic individuals. These findings provide a genetic framework for understanding HCC disparities and support future mechanistic and epidemiologic studies in this high-risk population.

## Simple Summary

In this study, we performed whole-exome sequencing on 719 Hispanic individuals (455 HCC cases) and conducted a series of rare variant analyses. We identified 29 rare deleterious variants in known HCC genes that are involved in lipid metabolism, immune regulation, and Wnt signaling. These variants are enriched in Hispanics, highlighting population-specific genetic susceptibility to HCC.

## 1. Introduction

Liver cancer is the sixth most common cancer and the third leading cause of cancer-related deaths worldwide [1]. Hepatocellular carcinoma (HCC) accounts for approximately 90% of primary liver cancers. Major risk factors for HCC include hepatitis B (HBV) and hepatitis C (HCV) virus infections, heavy alcohol consumption, and metabolic disorders [2]. Over the past decade, metabolic dysfunction-associated steatotic liver disease (MASLD), with and without alcohol use, has become an increasingly dominant driver of new HCC cases [3,4]. However, these risk factors explain a variable and low fraction (~10–~45%) of the overall HCC burden, and most individuals with these risk factors do not develop HCC [5]. Evidence of family clustering [6] and synergistic effects among alcohol consumption, metabolic disorders, and genetic predisposition suggest that inherited susceptibility plays a critical role in HCC risk [7].

Hispanic Americans, the largest and fastest-growing minoritized population in the United States, have the highest HCC age-adjusted incidence rates among all racial/ethnic groups [8]. Although this excess burden is partly attributable to a higher burden of metabolic disorders [9], alcoholic liver disease (ALD) and MASLD [10], HCC risk remains elevated even after adjusting for known risk factors, highlighting the potentially important contribution of genetic factors for HCC among Hispanic individuals [11].

Previous genome-wide association studies (GWAS) have identified multiple susceptibility loci and genes for HCC. These include common variants in genes such as *Myelin Associated Oligodendrocyte Basic Protein* (*MOBP*), *MAU2 Sister Chromatid Cohesion Factor* (*MAU2*) [12], *Patatin-like Domain 3*, *1-acylglycerol-3-phosphate O-Acyltransferase* (*PNPLA3*) [13], *Transmembrane 6 Superfamily Member 2* (*TM6SF2*) [14], *Human Leukocyte Antigen*, *class II DQ beta 1* (*HLA-DQB1)* [15], *class I polypeptide-related sequence A* (*MICA*) [16], *Telomerase Reverse Transcriptase* (*TERT*) [17], *Wnt Family Member 3A* (*WNT3A*), *Wnt Family Member 9A* (*WNT9A*) [18], *Complement C2* (*C2*) [19] and *Sorting and Assembly Machinery component* (*SAMM50*) [20]. However, these GWAS were conducted in mostly European and East Asian populations. As a result, the genetic architecture of HCC in Hispanic populations remains poorly characterized. Moreover, these GWAS studies focused on common variants (minor allele frequency [MAF] > 5%), which are predominantly located in non-coding regions and generally confer a small effect on HCC risk [13,14]. Given that these common variants explain only a limited proportion of HCC heritability (<30% [17,21]), particularly in ancestrally diverse populations, there is a critical need for studies focused on Hispanic groups.

Rare (MAF < 1%) and low-frequency (1% < MAF < 5%), coding variants have the great potential to explain the missing heritability [22], and often have a stronger effect size [23], as demonstrated for *BRCA1/2* in breast cancer [24]. Yet, rare variants have not been systematically studied in HCC, and few studies involved Hispanic or Latin American populations. One prior effort, conducted in a Mexican cohort, evaluated six previously reported common variants in *PNPLA3*, *Glucokinase Regulator* (*GCKR*) and *Membrane Bound Acylglycerophosphatidylinositol O-acyltransferase MBOAT7* (*MBOAT7*) with HCC risk [25]. However, this study did not assess rare coding variation and was limited to only three known loci. Thus, despite this initial work, systematic investigation of rare germline variants contributing to HCC risk in Hispanic individuals is still lacking, leaving a major gap in understanding population-specific genetic susceptibility.

In high-throughput sequencing-based rare variant association analyses, the statistical power is often limited due to the low-to-rare allele frequencies of individual variants [26]. However, genes implicated by common causal variants may also harbor rare variants that contribute to disease susceptibility [23,27], a pattern that demonstrated in liver cirrhosis [28]. Thus, focusing on HCC-associated genes may increase statistical power in rare variant analysis. In this study, we performed whole-exome sequencing for 719 Hispanic individuals and examined rare deleterious variants within genes previously implicated in HCC. We hypothesized that rare deleterious variants in key hepatic pathways contribute to HCC susceptibility in Hispanic individuals and that their effects are larger in this high-risk population.

## 2. Materials and Methods

### 2.1. Study Participants and Measurements

We conducted a case–control study of HCC among Hispanic individuals in Texas. HCC cases were recruited from clinical sites in Texas as part of the Texas Hispanic HCC collaborative. HCC diagnoses were confirmed by the tumor board at each facility using the American Association for the Study of Liver Diseases (AASLD) criteria, which includes histological and/or radiological diagnosis using characteristic appearance (arterial enhancement and delayed washout) on triple-phase computed tomography (CT) or magnetic resonance imaging (MRI). Internal controls were selected from the Starr County cohort study [29], a population-based cohort of individuals from Starr County, Texas, and had no cancer history. Demographic and clinical variables, such as age (at diagnosis for HCC cases or enrollment for controls), sex, and self-reported race/ethnicity, were obtained from medical records or the study questionnaire. Based on the clinical assessment of the treating physician, HCC etiology was classified as: (i) HCV, if the participant had a positive HCV RNA test or achieved sustained virological response at diagnosis; (ii) HBV, if hepatitis B surface antigen was positive; (iii) ALD based on a clinician-recorded diagnosis of ALD and self-reported current or former heavy alcohol use (≥8 drinks/week for women or ≥15 drinks/week for men); or (iv) MASLD requiring documented hepatic steatosis on liver histology or imaging in the absence of viral hepatitis, ALD, or other clinician-documented etiologies. Viral hepatitis was defined as either HCV or HBV.

### 2.2. Candidate Gene Selection

We selected genes that had been previously associated with HCC (Experimental Factor Ontology [EFO_0000182]) or MASLD (formerly NAFLD, EFO_0003095) through the GWAS Catalog [30] (accessed 4 August 2025). Additional candidate genes were identified from published GWAS or germline mutation studies identified through a literature review in PubMed. The final curated 86 candidate genes and supporting references are provided in Supplementary Table S1.

### 2.3. Whole-Exome Sequencing

Whole-exome sequencing of HCC cases and internal controls was performed at the Human Genome Sequencing Center (HGSC) at Baylor College of Medicine. Genomic DNA was extracted from peripheral blood samples that passed intake quality control using the Quant-iT™ PicoGreen^®^ dsDNA Assay Kit (Cat. #P7589, Thermo Fisher Scientific, Waltham, MA, USA). Libraries were prepared using NEB End Repair/dA-tailing and Ligation modules (Cat. # E6050L & E6053L, New England Biolabs, Ipswich, MA, USA) with 500 ng of genomic DNA input. Briefly, DNA samples were fragmented by sonication on a Covaris E220 instrument (Covaris, Woburn, MA, USA) in a 96-well format. Paired-end pre-capture libraries were generated using a fully automated workflow on Beckman Coulter Biomek FXp dual-arm robotic systems (Beckman Coulter, Inc., Stockholm, Sweden). Library preparation included DNA end repair, 3′-adenylation, and ligation to Illumina dual-indexed multiplexing adapters (Cat. #20022370, Illumina, Inc., San Diego, CA, USA). Adapter-ligated DNA fragments were subsequently PCR-amplified using KAPA HiFi DNA polymerase (Cat. #KK2612, Roche, Indianapolis, IN, USA) with primers complementary to the adapter sequences. For exome enrichment, pre-capture libraries were pooled and hybridized to HGSC-ClinExD probes (Twist Bioscience, South San Francisco, CA, USA, custom design), which target the GENCODE and RefSeq gene models and cover 35.9 Mb across approximately 20,000 genes. Post-capture libraries were subsequently combined into a single 90-plex pool for sequencing on one Illumina NovaSeq X lane (Illumina, Inc., San Diego, CA, USA) to generate 150 bp paired-end sequencing reads. Real-Time Analysis software (version 4.6.7) was used to monitor run performance, including cluster density, signal intensity, and phasing/pre-phasing metrics. Whole-exome sequencing was performed once for each individual, and no technical replicate sequencing was conducted. The sequencing coverage and quality statistics for each sample are summarized in Supplementary Table S2.

### 2.4. Alignment, Variant Calling, and Quality Control

Raw sequencing reads were aligned to the human reference genome (GRCh38) using BWA-MEM (version 0.7.15) [31]. Post-alignment processing, including base quality score recalibration and indel realignment, was performed using the Genome Analysis Toolkit (GATK, version 3.6.0) [32]. Single Nucleotide Variants (SNVs), short insertions or deletions (indels), were called using GATK HaplotypeCaller followed by joint genotyping with GenotypeGVCFs. Variant-level quality control steps included: (1) retaining variants with call rates ≥ 85%; (2) excluding variants with quality score < 30, depth < 10 or allelic balance < 2.0 for heterozygous calls; (3) excluding long indels (≥22 bp) and singleton variants; and (4) removing low-complexity repeats, segmental duplications and low-confidence variants that result from mapping errors, strand bias, weak exon conservation, and noisy background. All retained variants were visualized in Integrative Genome Viewer [33] to verify read depth and the pile-up pattern supporting each variant. Sample-level quality control steps included excluding samples with principal component analysis outliers, abnormal heterozygosity rate, sex discordance, <95% sample completion rates, or unexpected relatedness (defined as identity-by-state > 10%).

### 2.5. Variant Annotation and Filtering Process

**Figure 1:**
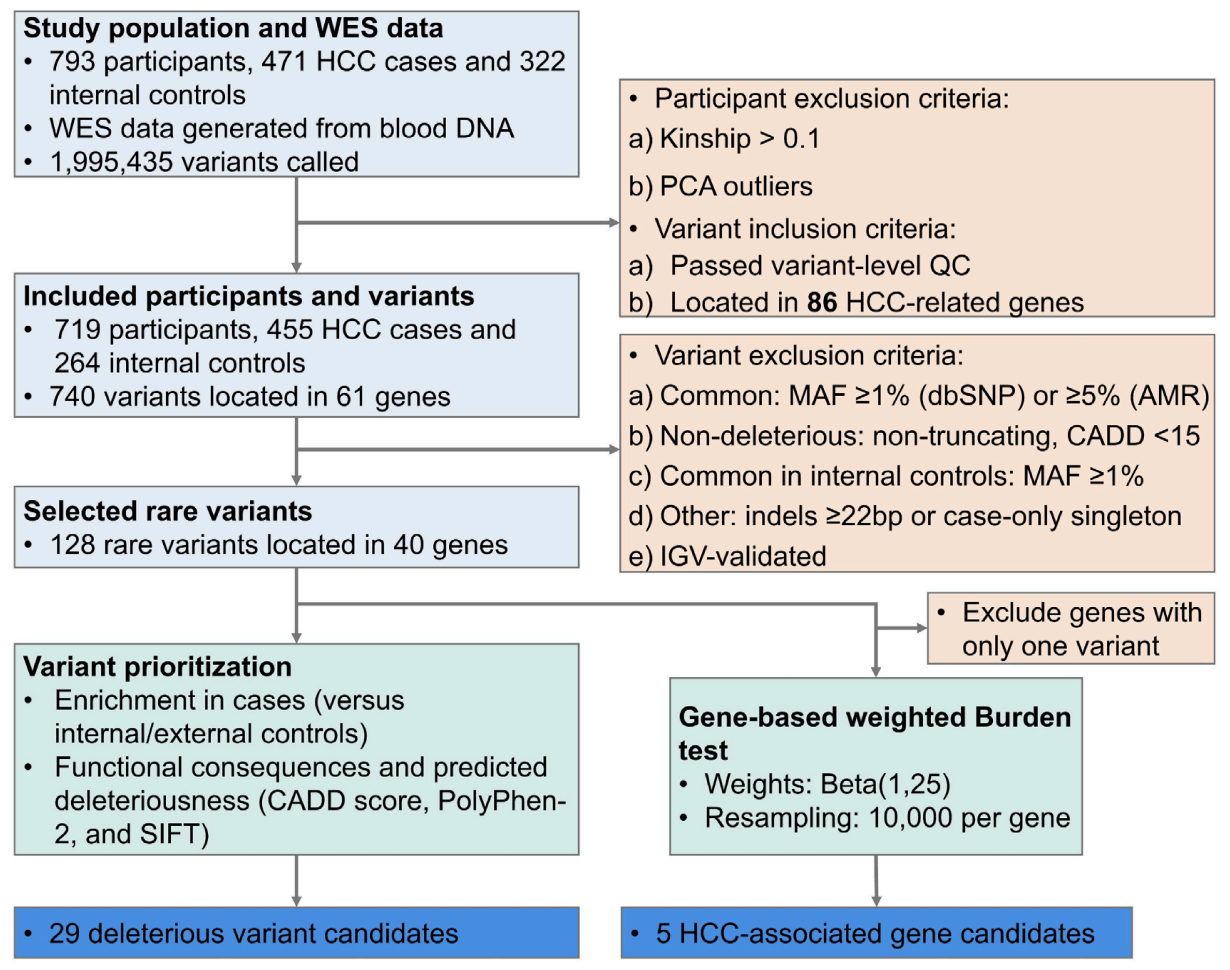
Study design and analytical workflow. WES, whole-exome sequencing; HCC, hepatocellular carcinoma; MAF, minor allele frequency; AMR, Admixed American from gnomAD; CADD, Combined Annotation Dependent Depletion; PolyPhen-2: Polymorphism Phenotyping v2; SIFT: Sorting Intolerant From Tolerant.

We focused on the variants mapped on the 86 candidate genes. Variants were annotated using Ensembl Variant Effect Predictor [34] (version 114) and filtered based on MAF and functionality as follows (Figure 1): (1) variants with MAF ≥ 1% in UCSC dbSNP tracks (build 155) were excluded; (2) common variants with MAF ≥ 5% in the Admixed American (AMR, *n* = 30,019) population of Genome Aggregation Database [35] (gnomAD, v4.1.0) were excluded to avoid variants that are rare in the general population but with low or common MAF in Hispanic ancestry groups. This genetic ancestry-defined AMR population includes a substantial proportion (41.76%) of individuals who self-identified as Mexican, Costa Rican, or Latino, making it an appropriate reference population for variant filtering and downstream analyses; (3) variants with MAF ≥ 1% among internal controls were further excluded to eliminate cohort-specific common variants; and (4) variants with a scaled Combined Annotation Dependent Depletion (CADD) [36] score ≥ 15 were retained.

### 2.6. Variant Prioritization

To streamline downstream analyses, we implemented an integrated variant prioritization framework combining functional consequences, in silico deleteriousness, and CADD score. Variants were first grouped into three tiers: (i) high—protein-truncating LoF variants (such as frameshift or stop-/gain-loss) and missense variants predicted damaging by both Polymorphism Phenotyping v2 (PolyPhen-2) and Sorting Intolerant From Tolerant (SIFT) [37,38]; (ii) medium—missense variants predicted damaging by either tool; and (iii) low—missense variants not predicted damaging but retained based on rarity and CADD. Across tiers, variants with CADD ≥ 15 (top ~3% deleterious in the human genome) were retained, except protein-truncating variants, which were included regardless. Variants were then ranked hierarchically by functional consequence (truncating > missense), in silico predicted deleteriousness tier (high > medium > low), and CADD score (higher prioritized). This integrated approach ensured that variants most likely to disrupt gene function were consistently prioritized for downstream analyses, while integrating functional and computational evidence.

### 2.7. Single-Variant Tests

MAFs were calculated separately for HCC cases and internal controls. We also included 30,019 individuals from the gnomAD AMR population as an external reference control. Single variant allelic-association tests were performed using Fisher’s exact test for two comparisons: (1) HCC cases versus internal controls (from the Starr County cohort study), and (2) HCC cases versus external gnomAD AMR population controls. Odds ratios (ORs) and 95% confidence intervals (CIs) were computed, and the false discovery rate (FDR) method was applied for multiple testing correction [39]. Stratified analysis was performed to compare the distribution of liver disease etiologies among HCC cases for each individual variant using Fisher’s exact tests. Statistical power for each single-variant comparison was calculated for Fisher’s exact test using the observed allele counts and MAFs in HCC cases, internal controls, and external gnomAD AMR controls. The estimated power for each comparison is summarized in Supplementary Table S3. As a sensitivity analysis, Firth’s penalized logistic regression was additionally performed for comparisons between HCC cases and internal controls, with adjustment for sex and the top three principal components generated by PLINK 2.0 [40] to adjust for population stratification.

### 2.8. Burden Test of Multiple Rare Deleterious Variants

Gene-based burden tests were performed for genes containing ≥ 2 rare variants using the Sequence Kernel Association Test [41] (SKAT version 2.2.5) R package to evaluate the cumulative effects of rare variants on HCC risk. The weighted burden test first collapses all included variants within a gene with their respective pre-assigned weights into a single burden score and then tests its association with HCC in analyses comparing HCC cases versus internal controls and adjusted for sex. The default Beta (1,25) probability distribution function was used for the weight in the models, which places greater weight on rarer variants. To ensure precise *p*-value estimation, 10,000 resampling iterations were performed. Sex was included as a covariate in all gene-based burden tests. Multiple testing correction was performed using the FDR method.

### 2.9. Dose-Effect Analysis

Dose–response effects of rare candidate variants on HCC risk were evaluated by comparing variant carriers and non-carriers in HCC cases and our internal controls using logistic regression models. We fitted logistic regression models for males and females combined and separately, treating the number of prioritized variants as a continuous predictor to estimate the change in HCC risk per additional rare variant carried. The Cochran–Armitage test [42] was used to test for trend.

## 3. Results

### 3.1. Overview of Study Cohort and Baseline Characteristics

After quality control, a total of 719 self-reported Hispanic participants were included in the analysis, comprising 455 HCC cases and 264 internal controls (Table 1). Among the HCC cases, 66.4% were male. The leading HCC etiologies were viral hepatitis (29.9%), ALD (23.1%), and MASLD (22.4%).

### 3.2. Rare Deleterious Variants Enriched in HCC Cases

From 1,995,435 variants detected in 719 Hispanic individuals, we selected 128 rare, potentially functional variants mapped to the 40 candidate genes, including missense (*n* = 112), frameshift (*n* = 7), splicing (*n* = 5), start-loss (*n* = 3) and stop-gain (*n* = 1) variants. Among these, 126 variants had CADD ≥ 15, except one frameshift and one start-loss variant.

We further evaluated 29 prioritized variants (located in 16 genes) that showed enrichment in HCC cases compared to external gnomAD AMR controls (nominal *p*-value < 0.05) by single variant allelic association test (Table 2). Nine of these variants remained statistically significant after FDR correction. Among these 29 variants, two were frameshift variants and the rest were missense variants. Based on deleteriousness levels, five variants were classified as high, six as medium, and 18 as low. Among the five highly deleterious variants, two were protein-truncating variants located in *HLA-DRB1*, and three were missense variants located in *Oncostatin M Receptor* (*OSMR*, *n* = 2) and *TM6SF2* (*n* = 1). The high deleteriousness variant *TM6SF2* R138W also has the highest CADD score, followed by *WNT9A* R357H. *TM6SF2* R138W also has a relatively higher allele frequency compared to other variants in HCC cases and the gnomAD AMR population. Out of the 29 variants, 23 showed higher allele frequencies in the gnomAD AMR compared with East Asian (EAS) and Non-Finnish European (NFE) populations.

In analyses comparing HCC cases with internal controls, we were unable to estimate ORs and 95% CIs for most variants as all internal controls were non-carriers. Despite this, we observed that *TM6SF2* R138W was significantly enriched and carriers had approximately 10-fold increased risk of HCC (carriers vs. non-carriers, OR 10.62, 95% CI: 1.67–443.08). When compared with the gnomAD AMR population, nine variants remained significantly associated with HCC risk after multiple testing corrections (Table 2). Among these nine, *C2* I484V exhibited the largest effect size (OR 26.43, 95% CI: 2.51–161.78) and *TM6SF2* R138W showed a moderate effect size (OR 2.66, 95% CI: 1.55–4.28). In addition, the allele frequency of *TM6SF2* R138W was more than two-fold higher in cases (allele frequency: 1.98%) than in the gnomAD AMR controls (allele frequency: 0.75%). As a sensitivity analysis, Firth’s penalized logistic regression yielded largely consistent effect estimates with those obtained from Fisher’s exact test, although confidence intervals remained wide because of the rarity of the variants (Supplementary Table S4).

**Figure 2:**
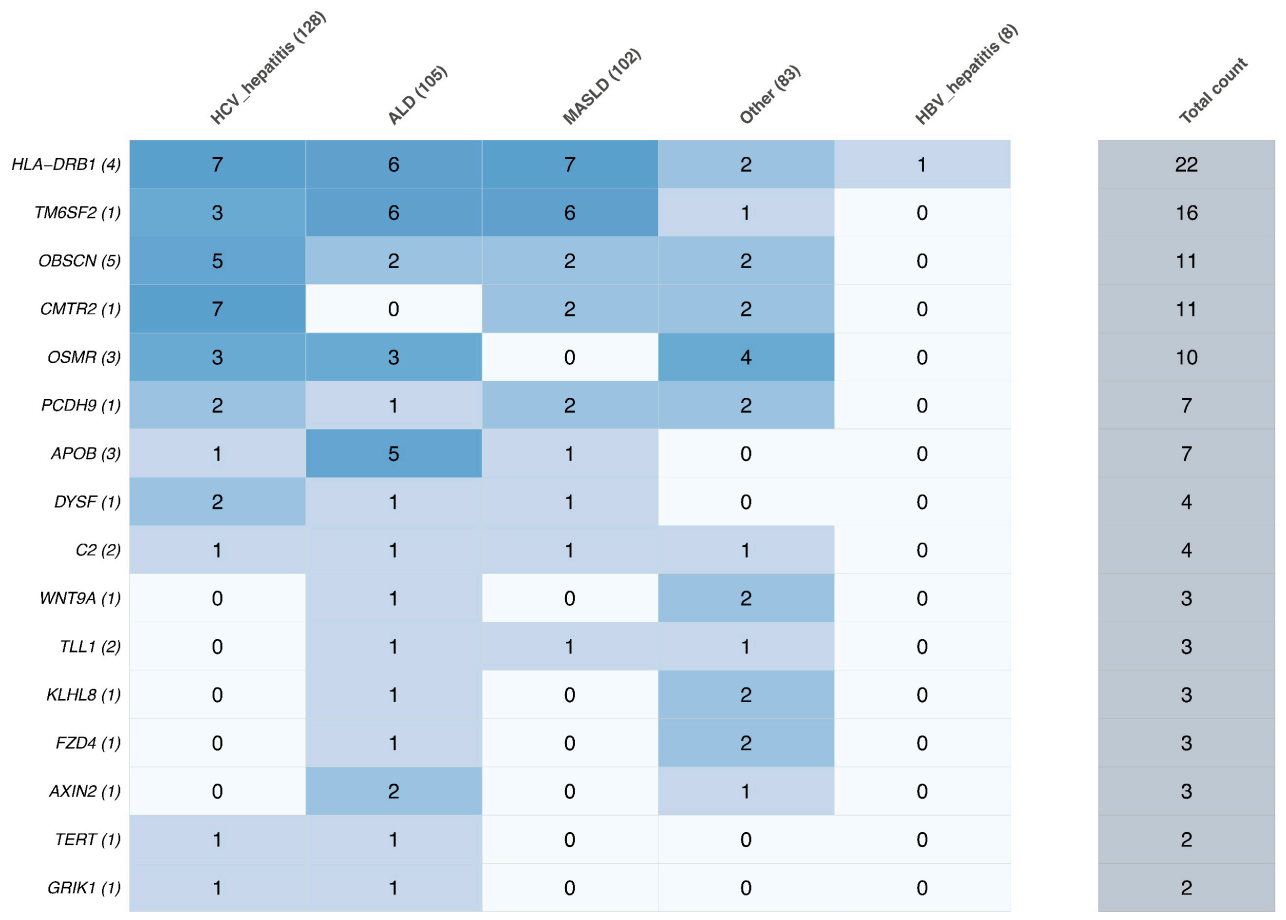
Distribution of prioritized rare variants by gene across etiologies. Each row represents a gene. The number in parentheses after each gene name indicates the number of prioritized variants identified in that gene. Each column represents an HCC etiology. The number in parentheses after each etiology indicates the total number of HCC cases in that etiology group. Values in each cell represent the number of variant occurrences observed for a given gene within each etiology. The last column shows the total number of variant occurrences across all HCC etiologies. Darker blue shading indicates a higher number of variant occurrences within each cell.

We performed exploratory analyses to examine the distribution of prioritized variants across different HCC etiologies. The frequency of the 29 prioritized variants appeared to differ among HCC cases by etiology (Figure 2). Variants in *HLA-DRB1*, *OSMR* and *C2* genes were observed among cases with viral hepatitis, ALD, and MASLD etiologies, while variants in *TM6SF2* and *APOB* were more frequently detected in ALD and MASLD-related HCC. In particular, *TM6SF2* was enriched among cases with ALD- or MASLD-related HCC, compared to other etiologies (OR 3.30, 95% CI: 0.98–14.27, nominal *p* = 0.040), supporting a potential etiology-specific role.

### 3.3. Gene-Level Burden Analysis Supported Cumulative Rare-Variant Effects

In the gene burden tests, a total of 23 genes containing at least two rare variants were analyzed. Of these, five genes were significantly associated with HCC (*p* < 0.05, Table 3), comprising 43 ultra-rare and nine rare variants. *TM6SF2* remained significantly associated with HCC risk after multiple testing corrections. Among these five genes, *TM6SF2* and *HLA-DRB1* overlapped with strong single-variant association signals (FDR-adjusted *p*-value < 0.05) while *Obscurin, Cytoskeletal Calmodulin and Titin-interacting RhoGEF* (*OBSCN*) and *OSMR* overlapped with moderate single-variant association signals (nominal *p*-value < 0.05, Table 2). Notably, rare variants in *TM6SF2* showed a large dose effect (OR 15.29, 95% CI: 2.06–113.51), and rare variants in *HLA-DRB1* showed a moderate dose effect (OR 2.50, 95% CI: 1.14–5.50).

### 3.4. HCC Risk Increased with the Number of Rare Deleterious Variants Carried

To evaluate whether the cumulative burden of rare variants contributed to increased HCC risk, we examined the association between the number of variants and HCC risk. When all 29 prioritized variants were considered, we observed a strong and statistically significant cumulative trend (OR 3.84, 95% CI: 2.40–6.53, Cochran–Armitage test, *p*-value = 1.56 × 10^−8^, Table 4). This trend was also observed when analyzed stratifying by sex (Supplementary Figure S2). Among all participants, 592 individuals were non-carriers of these 29 variants, whereas 127 individuals carried at least one variant (108 HCC cases and 19 internal controls, Supplementary Figure S1A). Among these 108 HCC carriers, most harbored a single variant, while 10 cases carried two variants and one HCC carried three variants (Supplementary Figure S1B). Among carriers of *TM6SF2* and *APOB*, co-occurring variants were observed in *C2*, *HLA-DRB1*, *OBSCN*, *OSMR* and *TERT*. When the analysis was restricted to the five variants classified as highly deleterious, a significant dose-effect trend was also observed, with a larger estimated effect size compared to the full set of 29 variants (OR 10.00, 95% CI: 3.03–61.85 versus OR 3.84, 95% CI: 2.40–6.53, Supplementary Figure S2).

## 4. Discussion

In this study, we demonstrate that rare deleterious germline variants are enriched in Hispanic HCC cases and exhibit effect sizes that far exceed those of common variants reported from HCC GWAS among European and Asian populations. We highlighted four variants, *TM6SF2* R138W, *APOB* I3721T, *HLA-DRB1* W38X, and *C2* I484V which represent major biological pathways that were markedly enriched in HCC cases, suggesting that rare coding variants may contribute to HCC susceptibility in Hispanic individuals (Supplementary Figure S3). These findings indicate population-specific genetic architecture of HCC, with certain rare variants exerting stronger effects in Hispanic individuals than previously observed in European or East Asian cohorts. These findings align with the disproportionate HCC burden and its etiologic features reported in Hispanic individuals, including a higher prevalence of metabolic syndrome [43], as well as different underlying etiologic patterns for HCC [44].

Our study extended prior findings on *TM6SF2*. Our group previously identified the common variants E167K as a susceptibility locus for HCC in populations of European descent in North American [12]. In the present study, we identified a rare variant, R138W, with a larger observed effect size (OR 2.66) in Hispanic individuals than that previously reported for the common E167K variant in European populations (OR 1.49) [12]. However, this comparison should be interpreted with caution because rare and common variants have different statistical properties, and effect estimates for rare variants may be inflated due to the winner’s curse. In exploratory analysis, R138W appeared to be enriched among ALD- or MASLD-related HCC cases. This observation suggests that this variant may modify risk in the context of alcohol-associated liver injury or metabolic dysregulation, consistent with the established role of *TM6SF2* in hepatic lipid metabolism. With an MAF of ~0.94% in the gnomAD AMR population, this variant may affect a non-trivial population-level risk of Hispanic individuals. Given growing evidence that rare variants improve risk stratification and refining disease diagnosis, incorporating R138W into prediction models for HCC arising from ALD- and MASLD-related cirrhosis may enhance risk discrimination in high-risk Hispanic populations.

Although the candidate genes included in this study were selected based on prior evidence of association with HCC, only a subset showed clear enrichment signals in our Hispanic HCC cohort, whereas many previously implicated genes did not. The enrichment of rare deleterious variants in lipid metabolism genes (i.e., *TM6SF2* and *APOB*) aligns with the high burden of MASLD and ALD in Hispanic populations and suggests that metabolic pathways may play a disproportionately large role in inherited HCC susceptibility in this group [43]. Rare variants in immune response genes, including *HLA-DRB1* and *C2*, also showed enrichment. *HLA-DRB1* has been implicated in susceptibility to several malignancies [45], supporting a hypothesis of immune-related genetic variation having a substantial impact on carcinogenesis. Given the high polymorphism and functional diversity of *HLA* loci, these findings warrant cautious interpretation but may reflect ancestry-specific immune modulation relevant to chronic liver injury and carcinogenesis.

Across analytical approaches, lipid metabolism and immune pathways were consistently identified in single-variant, gene-based burden, and dose–response analyses, reinforcing their robustness. In dose–response analysis, the five variants classified as highly deleterious were all mapped to these two pathways, further supporting their contribution to HCC susceptibility. Although *OBSCN* showed a strong association in the burden test, a few rare variants in this gene were identified in the single-variant analysis. Given that *OBSCN* is among the largest genes in the genome, it is more likely to accumulate rare variants by chance. Therefore, its association in the burden analysis may partly reflect gene size rather than a true biological contribution to HCC and should be interpreted with caution.

The main strength of our study is that it provides the first exome-sequencing data on HCC of Hispanic individuals in the US, addressing a population at high HCC risk, with high genetic diversity but limited prior genomic characterization. Previous work by Alejandro et al. examined only six known common variants in lipid metabolism genes and reported modest effect sizes (OR ~2.2) for three missense and one intronic variant [25]. In contrast, our study systematically evaluated rare coding variants across 86 genes, offering an initial basis for examining Hispanic-specific variation in HCC. The second major strength is our comprehensive etiological profiling of HCC cases (HBV, HCV, ALD, MASLD and other etiologies), which enhances interpretation of genetic findings. Also, because reliance on external controls alone can introduce confounding from platform differences, batch effects, and ancestry mismatch, we included internal controls drawn from the same geographic regions, and processed and sequenced alongside cases. This design improves geographic and ancestral comparability, reduces technical artifacts, and strengthens confidence in our rare variant filtering and discoveries.

Several limitations should be acknowledged. First, the small sample size limited our statistical power for whole-exome-wide discovery. To mitigate this, we focused on genes previously identified as HCC susceptibility genes from European and East Asian populations to improve statistical power. In addition, residual population stratification may also have influenced the observed associations because rare variants often exhibit different population stratification patterns compared with common variants [46]. Therefore, given the genetic heterogeneity among Hispanics across the US [47], validation in larger and independent cohorts representing diverse Hispanic ancestral backgrounds beyond our primarily Texas-based cohort will be necessary. Second, functional validation of *TM6SF2* R138W and other prioritized variants is needed to elucidate their underlying biological mechanisms. Future functional studies could use human hepatocyte cell lines and knock-in mouse models to validate the functional consequences of *TM6SF2* R138W on hepatic lipid metabolism and its downstream biological effects [48,49]. Third, the inclusion of affected or at-risk individuals among controls may have attenuated effect estimates. Precursor and related conditions, such as MASLD, ALD and cirrhosis, may also be present among the gnomAD controls, potentially leading to underestimation of true associations.

## 5. Conclusions

Our findings provide evidence that rare deleterious variants, especially in lipid metabolism and immune response, may contribute to increased HCC risk in Hispanic individuals, and suggest population-specific genetic architecture of HCC. The enrichment of variants such as *TM6SF2* R138W and the observed cumulative burden pattern highlighted the interplay between HCC risk and metabolic or environmental factors in this high-risk population. Future integration of coding, regulatory, environmental, and mechanistic data will be required to fully delineate the characteristics of high-risk subgroups and translate these findings into precision prevention strategies for Hispanic populations.

## Figures and Tables

**Table 1:** Baseline characteristics of study participants and gnomAD AMR reference population.

Characteristic ^a^	Cases (*n* = 455)	Controls (*n* = 264)	gnomAD AMR (*n* = 30,019)
Sex (Male, %)	302 (66.4)	89 (33.7)	13,775 (45.9)
Age (mean, SD)	60.6 (9.50)	48.3 (7.67)	N.A.
HCC etiology (%)			
Viral hepatitis B or C	136 (29.9)	-	-
Hepatitis C	128 (28.1)		
Hepatitis B	8 (1.8)		
ALD	105 (23.1)	-	-
MASLD	102 (22.4)	-	-
Other	83 (18.2)	-	-

Abbreviations: gnomAD, Genome Aggregation Database; AMR, Admixed American; N.A., not available; ALD, alcoholic liver disease; MASLD, metabolic dysfunction-associated steatotic liver disease. ^a^ Missing values (cases/controls): age (16/0); cirrhosis (29/-); HCC etiology (29/-).

**Table 2:** Prioritized 29 rare deleterious variant candidates *.

Gene	Variant ^a^	Deleterious Level ^b^	CADD ^c^	MAF % ^d^		Minor Allele Count	HCC Cases vs. gnomAD AMR	
				Case/Control	AMR/EAS/NFE/Overall ^e^	Case/Control/AMR	OR (95% CI)	FDR *p* Value ^f^
*HLA-DRB1*	c.101-1G>A	High	25	0.39/0	0.071/0.0065/0.14/0.14	3/0/22	5.45 (1.04–18.21)	0.1070
*HLA-DRB1*	**W38X**	High	21.6	0.73/0.21	0.049/0.019/0.02/0.024	6/1/18	15.13 (4.91–39.90)	**0.0003**
*TM6SF2*	**R138W**	High	29.8	1.98/0.19	0.75/0.065/0.00068/0.031	18/1/452	2.66 (1.55–4.28)	**0.0061**
*OSMR*	V436D	High	23.5	0.55/0	0.16/0/0.43/0.34	5/0/98	3.38 (1.07–8.18)	0.1004
*OSMR*	D262A	High	22.1	0.22/0	0.032/0/0.04/0.031	2/0/19	6.95 (0.78–28.91)	0.1489
*WNT9A*	R357H	Medium	28.6	0.44/0	0.093/0.02/0.2/0.16	4/0/56	4.72 (1.24–12.82)	0.0974
*AXIN2*	P245S	Medium	23.5	0.33/0	0.053/0/0.063/0.052	3/0/32	6.20 (1.21–19.87)	0.0976
*OBSCN*	T6187M	Medium	23.3	0.44/0.76	0.15/0/0.031/0.031	4/4/88	3.01 (0.80–8.00)	0.1782
*OBSCN*	V5337M	Medium	22.5	0.22/0	0.017/0.0045/0.001/0.0017	2/0/10	12.88 (1.37–60.53)	0.0976
*OBSCN*	G3945R	Medium	20.6	0.33/0.57	0.058/0/0.0064/0.0079	3/3/35	5.67 (1.11–18.02)	0.1004
*APOB*	**I3721T**	Medium	20.1	0.33/0	0.033/0/0/0.0012	3/0/20	9.92 (1.88–33.53)	**0.0467**
*GRIK1*	**E821K**	Low	24.7	0.22/0	0.0083/0/0.0025/0.0025	2/0/5	26.41 (2.51–161.69)	**0.0467**
*DYSF*	D1477G	Low	24.1	0.44/0	0.13/0/0.0025/0.14	4/0/77	3.43 (0.91–9.18)	0.1371
*C2*	T184M	Low	23.1	0.22/0	0.018/0/0.004/0.0038	2/0/11	12.02 (1.29–55.16)	0.0976
*TLL1*	N84I	Low	22.8	0.22/0.19	0.03/0.0022/0/0.0012	2/1/18	7.32 (0.82–30.64)	0.1412
*TERT*	A730T	Low	20.7	0.22/0	0.022/0/0.00093/0.0016	2/0/13	10.17 (1.11–44.99)	0.1020
*HLA-DRB1*	**Q260P**	Low	19.98	0.78/0	0.18/0.11/0.037/0.1	6/0/77	4.34 (1.54–9.93)	**0.0467**
*OBSCN*	R6892Q	Low	19.98	0.22/0	0.02/0/0.0019/0.0031	2/0/12	11.01 (1.20–49.61)	0.1004
*PCDH9*	S1209R	Low	19.24	0.77/0.19	0.25/0.0067/0.27/0.31	7/1/147	3.15 (1.24–6.68)	0.0808
*CMTR2*	S331P	Low	17.88	1.21/0.38	0.52/0.0022/0.00017/0.02	11/2/314	2.33 (1.15–4.24)	0.0886
*KLHL8*	**S178R**	Low	17.87	0.33/0	0.022/0/0/0.00081	3/0/13	15.26 (2.78–55.60)	**0.0274**
*APOB*	R778H	Low	17.75	0.22/0	0.025/0/0.00093/0.0017	2/0/15	8.81 (0.98–37.96)	0.1152
*TLL1*	A815D	Low	17.74	0.22/0	0.035/0.0022/0/0.0014	2/0/21	6.27 (0.71–25.70)	0.1716
*APOB*	D3907G	Low	17.61	0.22/0.19	0.025/0/0.0011/0.0077	2/1/15	8.81 (0.98–37.95)	0.1152
*OSMR*	K154T	Low	17.5	0.33/0	0.07/0/0/0.0026	3/0/42	4.72 (0.93–14.82)	0.1260
*C2*	**I484V**	Low	17.49	0.22/0.19	0.0083/0/0.0059/0.022	2/1/5	26.43 (2.51–161.78)	**0.0467**
*HLA-DRB1*	**M20T**	Low	16.82	1.26/0.66	0.086/0.084/0.055/0.067	10/3/34	14.93 (6.55–31.04)	**<0.0001**
*FZD4*	**V184L**	Low	15.25	0.33/0	0.023/0/0/0.00099	3/0/14	14.17 (2.61–50.95)	**0.0288**
*OBSCN*	S4397L	Low	15.2	0.22/0.19	0.02/0.011/0.00059/0.0017	2/1/12	10.96 (1.19–49.37)	0.1004

Abbreviations: CADD, Combined Annotation Dependent Depletion; MAF, minor allele frequency; AMR, Admixed American; EAS, East Asian; NFE, European (non-Finnish); OR, odds ratio; CI, confidence interval; FDR, false discovery rate. ^a^ The variant c.101-1G>A represents a splice acceptor variant and W38X denotes a stop-gain variant. Variants in bold indicate those significantly enriched in HCC cases compared with the gnomAD AMR population (FDR *p*-value < 0.05). ^b^ Deleteriousness levels were defined based on functional consequences. Protein-truncating variants and missense variants predicted as “probably damaging” by PolyPhen-2 and “deleterious” by SIFT were classified as “High”. Variants predicted as such by either tool were classified as “Medium”, and those predicted as neither were classified as “Low”. ^c^ CADD score reflects the predicted deleteriousness of variants in the human genome. Phred-scaled CADD scores of ≥10, ≥20, ≥30 correspond to the top 10%, 1%, and 0.1% most deleterious variants, respectively. ^d^ MAF reported in %. ^e^ AMR, EAS, NFE and overall MAF were retrieved from Genome Aggregation Database allele frequency. ^f^ *p*-values were adjusted for multiple testing using the FDR method. * Variants were selected using a nominal *p* < 0.05 in the comparison with the external gnomAD AMR controls. Variants shown in bold remained significant after FDR correction.

**Table 3:** Burden test results for 23 HCC-related genes with at least 2 rare variants.

Gene	N. Variant in MAF Bin ^a^		N. Variant Carriers Case/Control	OR (95% CI) ^b^	*p*-Value ^c^	FDR *p*-Value ^d^
	Ultra Rare 0.001–0.005	Low Frequency 0.005–0.0132				
*TM6SF2*	3	1	25/1	15.29 (2.06–113.51)	0.0004	0.0092
*OBSCN*	30	4	88/34	1.62 (1.06–2.49)	0.0115	0.1322
*MICA*	1	1	12/2	3.53 (0.78–15.90)	0.0252	0.1504
*HLA-DRB1*	5	2	33/8	2.50 (1.14–5.50)	0.0262	0.1504
*OSMR*	4	1	26/6	2.61 (1.06–6.42)	0.0453	0.2084
*MAU2*	2	0	4/0	5.27 (0.28–98.31)	0.0791	0.3032
*TERT*	2	0	5/0	6.46 (0.36–117.26)	0.0927	0.3044
*MBOAT7*	2	0	8/1	4.71 (0.59–37.84)	0.1260	0.3624
*DYSF*	5	2	29/10	1.73 (0.83–3.61)	0.1694	0.3903
*EVA1C*	2	0	8/1	4.71 (0.59–37.84)	0.1697	0.3903
*MTARC1*	2	0	9/2	2.64 (0.57–12.33)	0.2347	0.4898
*C2*	4	0	8/2	2.34 (0.49–11.12)	0.2762	0.4898
*MRPS11*	0	2	19/7	1.60 (0.66–3.86)	0.2768	0.4898
*KIF1B*	2	0	6/2	1.75 (0.35–8.74)	0.3139	0.5158
*FZD4*	1	1	9/2	2.64 (0.57–12.33)	0.3728	0.5587
*TLL1*	2	0	4/1	2.33 (0.26–20.98)	0.4075	0.5587
*SQSTM1*	3	0	8/3	1.56 (0.41–5.92)	0.4130	0.5587
*TRIM31*	1	2	7/7	0.57 (0.2–1.65)	0.5420	0.6611
*AXIN2*	3	0	8/6	0.77 (0.26–2.24)	0.5461	0.6611
*PCDH9*	4	1	14/7	1.17 (0.46–2.93)	0.7391	0.8300
*HLA-DPA1*	2	0	4/1	2.33 (0.26–20.98)	0.7578	0.8300
*VEPH1*	6	0	8/5	0.93 (0.3–2.86)	0.9123	0.9270
*APOB*	7	1	23/9	1.51 (0.69–3.31)	0.9270	0.9270

Abbreviations: MAF, minor allele frequency; OR, odds ratio; CI, confidence interval; FDR, false discovery rate. ^a^ MAFs were calculated within the study population (combined cases and controls), and therefore, some variants may have MAF > 0.01 in this dataset despite being classified as rare. ^b^ ORs and 95% CIs were estimated by comparing carriers (≥1 included variant in a given gene) with non-carriers. ^c^ *p*-values were calculated from gene-based weighted burden tests. ^d^ *p*-values were adjusted for multiple testing using the FDR method across 23 tests.

**Table 4:** Distribution of individuals by the number of deleterious variants carried.

Group	N. Variants	Case	Control	OR (95% CI) ^a^	*p*-Value
Non-Carriers	0	347	245	Ref 1.0	-
Carriers	1	97	19	3.84 (2.40–6.53)	1.19 × 10^−7^
	2	10	0		
	3	1	0		

Abbreviations: OR, odds ratio. ^a^ OR was estimated using logistic regression and represents the effect per additional variant, Cochran–Armitage trend test, *p* = 1.56 × 10^−8^.

## Data Availability

Data supporting the findings of this study are available within the article and its Supplementary Materials. The whole-exome sequencing data generated in this study contain individual-level human genomic information and are subject to institutional, ethical, legal, and participant consent restrictions designed to protect participant privacy. Data and supporting documentation will be made available through Baylor College of Medicine’s section of Digital Commons@TMC. Access to genomic data will be provided in accordance with applicable institutional review board, data use, and privacy requirements. Additional information supporting the findings of this study is available from the corresponding author upon reasonable request.

## Supplementary Materials

The following supporting information can be downloaded at: https://www.mdpi.com/article/10.3390/cancers18152428/s1, Figure S1A: Landscape of the 29 prioritized rare deleterious variants across 127 carriers, including 108 HCC cases (black) and 19 controls (blank white). Each column represents one participant. Upper 4 rows represent HCC status, demographic characteristics (age, sex) and etiology, and lower 29 rows represent variants in genes. Colored cells in gene rows indicate the presence of specific variants. For genes with more than 1 variants, colors distinguish the specific variants. HCC, Hepatocellular carcinoma; ALD, Alcoholic liver dis-ease; MASLD, Metabolic dysfunction-associated steatotic liver disease; HCV, Hepatitis C virus; Figure S1B: Landscape of variants of 11 individuals carrying at least two prioritized variants. Among these individuals, 9 have ALD or MASLD, and 1 has HCV hepatitis. Each column represents one participant. Upper 3 rows represent age, sex and etiology, and lower 19 rows represent variants in genes. HCC, Hepatocellular carcinoma; ALD, Alcoholic liver disease; MASLD, Metabolic dysfunc-tion-associated steatotic liver disease; HCV, Hepatitis C virus; Figure S2: Association between rare variant burden and HCC risk. Odds ratios (ORs) and 95% confidence intervals (CIs) for HCC risk per additional variant carried are shown for all 29 prioritized rare variants (top panel) and for the subset of five variants classified as high deleteriousness (bottom panel). Estimates were obtained from logistic regression models with the number of variants carried as a continuous predictor. Analyses were performed for all participants combined, and stratified by sex. The dashed vertical line indicates OR = 1; Figure S3: Flow chart of variant prioritization and representative biological pathways; Table S1: Curated list of candidate genes associated with HCC and supporting references; Table S2: Sequencing coverage and quality statistics; Table S3: Estimated statistical power of single-variant association comparison; Table S4: Comparison of odds ratios for the 29 prioritized variants estimated using Fisher’s exact test and Firth’s penalized logistic regression.

## Abbreviations

AASLD, American Association for the Study of Liver Diseases; ALD, alcoholic liver disease; AMR, Admixed American; C2, Complement C2; CADD, Combined Annotation Dependent Depletion; CI, confidence interval; CT, Computed Tomography; EAS, East Asian; FDR, false discovery rate; GATK, Genome Analysis Toolkit; GCKR, Glucokinase Regulator; gnomAD, Genome Aggregation Database; GWAS, genome-wide association studies; HBV, hepatitis B virus; HCC, hepatocellular carcinoma; HCV, hepatitis C virus; HGSC, Human Genome Sequencing Center; HLA-DQB1, human leukocyte antigen, class II DQ beta 1; MAF, minor allele frequency; MASLD, metabolic dysfunction-associated steatotic liver disease; MAU2, MAU2 sister chromatid cohesion factor; MBOAT7, membrane bound acylglycerophosphatidylinositol O-acyltransferase MBOAT7; MICA, human leukocyte antigen, class I polypeptide-related sequence A; MOBP, myelin associated oligodendrocyte basic protein; MRI, Magnetic Resonance Imaging; NAFLD, nonalcoholic fatty liver disease; NFE, Non-Finnish European; OR, odds ratio; OBSCN, obscurin, cytoskeletal calmodulin and titin-interacting RhoGEF; OSMR, oncostatin M receptor; PNPLA3, patatin-like domain 3, 1-acylglycerol-3-phosphate O-acyltransferase; PolyPhen-2, Polymorphism Phenotyping v2; SAMM50, sorting and assembly machinery component; SIFT, Sorting Intolerant From Tolerant; SKAT, Sequence Kernel Association Test; SNV, Single Nucleotide Variant; TERT, telomerase reverse transcriptase; TM6SF2, transmembrane 6 superfamily member 2; WNT3A, Wnt family member 3A; WNT9A, Wnt family member 9A.
