# Supplementary material for: Rare Germline Variants in Key Pathways Contribute to Hepatocellular Carcinoma Risk in Hispanic Individuals
Source: Cancers (Basel). 2026 Jul 28;18(15):2428. doi: 10.3390/cancers18152428 (PMC13464447; doi:10.3390/cancers18152428)
Supplement: Supplementary file 1 [file cancers-18-02428-s001.zip › Supplementary_Table_S3.pdf]

Supplementary Table S3. Estimated statistical power of single-variant association comparison

Variants	MAF %	Allele count	Power	
	Case / Control / AMR	Case / Control / AMR	vs Control	vs AMR
HLA-DRB1: c.101-1G>A	0.39 / 0 / 0.071	778 / 446 / 31018	0.0037	0.57
HLA-DRB1: W38X	0.73 / 0.21 / 0.049	818 / 476 / 36904	0.10	0.95
TM6SF2: R138W	1.98 / 0.19 / 0.75	910 / 528 / 60010	0.90	0.91
OSMR: V436D	0.55 / 0 / 0.16	910 / 528 / 60020	0.13	0.59
OSMR: D262A	0.22 / 0 / 0.032	910 / 528 / 60022	0.0011	0.54
WNT9A: R357H	0.44 / 0 / 0.093	910 / 528 / 59966	0.051	0.63
AXIN2: P245S	0.33 / 0 / 0.053	910 / 528 / 60022	0.012	0.59
OBSCN: T6187M	0.44 / 0.76 / 0.15	910 / 528 / 60004	0.094	0.47
OBSCN: V5337M	0.22 / 0 / 0.017	910 / 528 / 58470	0.0011	0.59
OBSCN: G3945R	0.33 / 0.57 / 0.058	910 / 528 / 60008	0.074	0.58
APOB: I3721T	0.33 / 0 / 0.033	910 / 528 / 60008	0.012	0.74
GRIK1: E821K	0.22 / 0 / 0.0083	910 / 528 / 59978	0.0011	0.63
DYSF: D1477G	0.44 / 0 / 0.13	910 / 528 / 59974	0.051	0.55
C2: T184M	0.22 / 0 / 0.018	910 / 528 / 60028	0.0011	0.59
TLL1: N84I	0.22 / 0.19 / 0.03	910 / 528 / 59868	0.012	0.56
TERT: A730T	0.22 / 0 / 0.022	910 / 528 / 60012	0.0011	0.59
HLA-DRB1: Q260P	0.78 / 0 / 0.18	768 / 456 / 42516	0.26	0.76
OBSCN: R6892Q	0.22 / 0 / 0.02	910 / 528 / 60016	0.0011	0.59
PCDH9: S1209R	0.77 / 0.19 / 0.25	910 / 528 / 59862	0.17	0.70
CMTR2: S331P	1.21 / 0.38 / 0.52	910 / 528 / 60024	0.27	0.67
KLHL8: S178R	0.33 / 0 / 0.022	910 / 528 / 60004	0.012	0.80
APOB: R778H	0.22 / 0 / 0.025	910 / 528 / 60022	0.0011	0.59
TLL1: A815D	0.22 / 0 / 0.035	910 / 528 / 59782	0.0011	0.50
APOB: D3907G	0.22 / 0.19 / 0.025	910 / 528 / 59996	0.012	0.59
OSMR: K154T	0.33 / 0 / 0.07	910 / 528 / 60004	0.012	0.56
C2: I484V	0.22 / 0.19 / 0.0083	910 / 528 / 60012	0.012	0.63
HLA-DRB1: M20T	1.26 / 0.66 / 0.086	792 / 454 / 39730	0.10	1.00
FZD4: V184L	0.33 / 0 / 0.023	910 / 528 / 59996	0.012	0.80
OBSCN: S4397L	0.22 / 0.19 / 0.02	910 / 528 / 59734	0.012	0.59

Abbreviations: MAF, Minor allele frequency; AMR, Admixed American
