# Supplementary material for: Rare Germline Variants in Key Pathways Contribute to Hepatocellular Carcinoma Risk in Hispanic Individuals
Source: Cancers (Basel). 2026 Jul 28;18(15):2428. doi: 10.3390/cancers18152428 (PMC13464447; doi:10.3390/cancers18152428)
Supplement: Supplementary file 1 [file cancers-18-02428-s001.zip › Supplementary_Table_S4.pdf]

Supplementary Table S4. Comparison of odds ratios for the 29 prioritized variants estimated using Fisher's exact test and Firth's penalized logistic regression

Variants	OR (95% CI)				
	HCC vs Controls				HCC vs AMR
	Fisher's exact test	Firth's panelized logistic regression			Fisher's exact test
		No adjustment	Adjust for sex	Adjust for sex and top3 PCs	
HLA-DRB1: c.101-1G>A	Inf (0.24-Inf)	4.05 (0.39-545.2)	4.24 (0.34-597.44)	4.48 (0.37-617.05)	5.45 (1.04-18.21)
HLA-DRB1: W38X	3.51 (0.42-161.67)	2.55 (0.53-24.58)	1.75 (0.34-17.7)	1.47 (0.28-14.79)	15.13 (4.91-39.90)
TM6SF2: R138W	10.62 (1.67-443.08)	7.43 (1.87-67.36)	8.26 (2-76.13)	5.31 (1.18-50.88)	2.66 (1.55-4.28)
OSMR: V436D	Inf (0.53-Inf)	6.46 (0.73-849.08)	3.31 (0.37-435.98)	4.99 (0.43-690.78)	3.38 (1.07-8.18)
OSMR: D262A	Inf (0.11-Inf)	2.92 (0.24-402.81)	5.79 (0.47-801.3)	60.08 (2.89-9000.1)	6.95 (0.78-28.91)
WNT9A: R357H	Inf (0.38-Inf)	5.27 (0.56-699.71)	4.71 (0.44-649.06)	11.91 (0.72-1759.67)	4.72 (1.24-12.82)
AXIN2: P245S	Inf (0.24-Inf)	4.09 (0.39-550.96)	2.09 (0.2-282.26)	0.83 (0.07-114.47)	6.20 (1.21-19.87)
OBSCN: T6187M	0.58 (0.11-3.12)	0.58 (0.15-2.25)	0.7 (0.16-2.92)	1.35 (0.2-8.94)	3.01 (0.80-8.00)
OBSCN: V5337M	Inf (0.11-Inf)	2.92 (0.24-402.81)	3.73 (0.25-537.25)	1.41 (0.11-195.76)	12.88 (1.37-60.53)
OBSCN: G3945R	0.58 (0.08-4.34)	0.58 (0.12-2.74)	0.58 (0.11-3.02)	1.44 (0.25-8.46)	5.67 (1.11-18.02)
APOB: I3721T	Inf (0.24-Inf)	4.09 (0.39-550.96)	2.09 (0.2-282.26)	0.8 (0.07-109.56)	9.92 (1.88-33.53)
GRIK1: E821K	Inf (0.11-Inf)	2.92 (0.24-402.81)	1.49 (0.12-206.13)	0.48 (0.04-67.43)	26.41 (2.51-161.69)
DYSF: D1477G	Inf (0.38-Inf)	5.27 (0.56-699.71)	6.71 (0.64-910.72)	2.45 (0.23-331.6)	3.43 (0.91-9.18)
C2: T184M	Inf (0.11-Inf)	2.92 (0.24-402.81)	1.49 (0.12-206.13)	0.61 (0.05-86.02)	12.02 (1.29-55.16)
TLL1: N84I	1.16 (0.06-68.59)	0.97 (0.13-10.62)	0.82 (0.1-10.15)	0.36 (0.03-5.03)	7.32 (0.82-30.64)
TERT: A730T	Inf (0.11-Inf)	2.92 (0.24-402.81)	3.73 (0.25-537.25)	2.29 (0.15-331.78)	10.17 (1.11-44.99)
HLA-DRB1: Q260P	Inf (0.7-Inf)	7.85 (0.92-1025.28)	3.97 (0.46-520.48)	1.53 (0.13-212.99)	4.34 (1.54-9.93)
OBSCN: R6892Q	Inf (0.11-Inf)	2.92 (0.24-402.81)	3.73 (0.25-537.25)	5.74 (0.39-828.8)	11.01 (1.20-49.61)
PCDH9: S1209R	4.08 (0.52-184.32)	2.94 (0.64-27.96)	2.43 (0.48-24.23)	1.44 (0.28-14.61)	3.15 (1.24-6.68)
CMTR2: S331P	3.22 (0.7-29.97)	2.72 (0.79-14.06)	2.42 (0.66-13.05)	1.59 (0.41-8.81)	2.33 (1.15-4.24)
KLHL8: S178R	Inf (0.24-Inf)	4.09 (0.39-550.96)	2.09 (0.2-282.26)	0.48 (0.04-66.19)	15.26 (2.78-55.60)
APOB: R778H	Inf (0.11-Inf)	2.92 (0.24-402.81)	3.73 (0.25-537.25)	1.37 (0.1-194.92)	8.81 (0.98-37.96)
TLL1: A815D	Inf (0.11-Inf)	2.92 (0.24-402.81)	3.73 (0.25-537.25)	0.87 (0.05-127.54)	6.27 (0.71-25.70)
APOB: D3907G	1.16 (0.06-68.59)	0.97 (0.13-10.62)	0.49 (0.06-5.41)	0.18 (0.02-2.26)	8.81 (0.98-37.95)
OSMR: K154T	Inf (0.24-Inf)	4.09 (0.39-550.96)	4.19 (0.34-588.57)	1.23 (0.09-177.25)	4.72 (0.93-14.82)
C2: I484V	1.16 (0.06-68.59)	0.97 (0.13-10.62)	1.34 (0.15-15.65)	0.85 (0.09-11.89)	26.43 (2.51-161.78)
HLA-DRB1: M20T	1.92 (0.49-10.92)	1.74 (0.56-6.96)	2.58 (0.79-10.62)	3.05 (0.82-14.01)	14.93 (6.55-31.04)
FZD4: V184L	Inf (0.24-Inf)	4.09 (0.39-550.96)	2.09 (0.2-282.26)	0.5 (0.05-68.13)	14.17 (2.61-50.95)
OBSCN: S4397L	1.16 (0.06-68.59)	0.97 (0.13-10.62)	1.34 (0.15-15.65)	0.74 (0.07-9.53)	10.96 (1.19-49.37)

Abbreviations: OR, Odds ratio; HCC Hepatocellular carcinoma; AMR, Admixed American; PC, Principal

component
